# Patient-specific modelling of pulmonary arterial hypertension: wall shear stress correlates with disease severity

**DOI:** 10.3389/fbioe.2025.1585345

**Published:** 2025-06-18

**Authors:** C. H. Armour, D. Gopalan, B. Statton, D. P. O’Regan, L. Howard, M. R. Wilkins, X. Y. Xu, A. Lawrie

**Affiliations:** ^1^ National Heart and Lung Institute, Imperial College London, London, United Kingdom; ^2^ Department of Chemical Engineering, Imperial College London, London, United Kingdom; ^3^ National Pulmonary Hypertension Service, Imperial College Healthcare NHS Trust, London, United Kingdom; ^4^ Medical Research Council, Laboratory of Medical Sciences, Imperial College London, London, United Kingdom

**Keywords:** pulmonary arterial hypertension, computational fluid dynamics, 4D-flow MRI, wall shear stress, non-invasive assessment, computational biomarker

## Abstract

**Introduction:**

Pulmonary arterial hypertension (PAH) requires an invasive right heart catheter (RHC) procedure for diagnosis. Patients can present with initial symptoms and interact with healthcare institutes for up to 3 years before referral for diagnosis. Thus, there is a great need to develop non-invasive tools, to better screen patients and improve early diagnosis rates.

**Methods:**

seven patients diagnosed and treated for PAH were included in this study. Patient-specific computational fluid dynamics (CFD) models were built for all patients, with all model parameters tuned using non-invasive imaging data, including CT, cardiac MR, echocardiogram, and 4D-flow MRI scans–crucially, a 3D inlet velocity profile was derived from 4D-flow MRI.

**Results:**

CFD models were quantitatively and qualitatively well matched with *in-vivo* 4D-flow hemodynamics. A linear correlation of R^2^ = 0.84 was found between CFD derived time-averaged wall shear stress (TAWSS) and RHC measured mean pulmonary pressure (key diagnostic value): low TAWSS correlated with high pressure.

**Conclusion:**

This study highlights TAWSS as a potential computational biomarker for PAH. The clinical use of TAWSS to diagnose and stratify PAH patients has the potential to greatly improve patient outcomes. Further work is ongoing to validate these findings in larger cohorts.

## 1 Introduction

Pulmonary arterial hypertension (PAH) is characterized by high blood pressure (>20 mmHg) in the pulmonary arteries (Simonneau et al., 2019). Current medical guidelines require an invasive right heart catheter (RHC) procedure to measure pulmonary arterial pressure (PAP) and diagnose PAH (Humbert et al., 2023). In the UK and Ireland, an RHC will be conducted at one of nine specialist pulmonary hypertension centres. However, studies have shown that PAH patients can present to local healthcare institutions up to 3 years before diagnosis with initial symptoms such as breathlessness (Kiely et al., 2019a). In the period between first presentation and referral for RHC these patients will continue to interact with the healthcare system through A&E visits, inpatient hospitalisations, outpatient consultations, etc., (Lawrie et al., 2024; Bergemann et al., 2018). Within this time, extensive data, including blood tests, clinical tests, and imaging studies, will be collected (Kiely et al., 2019a; 2019b). Despite a wealth of information available on these patients, and increasing awareness of PAH, there has been little improvement in time from symptom onset to diagnosis. Early diagnosis of PAH is known to improve PAH management and reduce healthcare costs (Lawrie et al., 2024). Thus, there is a great need to develop non-invasive methods to estimate PAP that can utilise the extensive data sets available on patients presenting continuously, to better identify those that are at risk of PAH.

Transthoracic echocardiogram (TTE) is one such non-invasive method from which PAP can be estimated. While TTE is cheap and can easily be performed bedside, several studies have reported its high potential for misdiagnosis - in particular, TTE performs poorly on patients with PAPs close to the diagnostic threshold of 20 mmHg (Testani et al., 2010; Fisher et al., 2009; Rich et al., 2011). 4D-flow MRI is a relatively newer non-invasive technology that captures 3D velocity fields in the scan region of interest at multiple time points within a cardiac cycle. This provides an extensive amount of highly valuable *in-vivo* hemodynamic data. Studies have identified 4D-flow MRI measured parameters including time-averaged wall shear stress (TAWSS) and vortical flow to correlate with disease severity (Schafer et al., 2016; Cerne et al., 2022; Barker et al., 2015; Reiter et al., 2020; Deux et al., 2022). However, exact magnitudes of velocity derivative parameters are known to be inaccurate when evaluated from 4D-flow directly due to the low spatial and temporal resolution of the scans (Miyazaki et al., 2017; Zhang et al., 2022), with spatial resolution typically in the region of 2.5 mm^3^ (Zilber et al., 2021), and temporal resolution approximately 0.04 s (equivalent to 25 time points in a cardiac cycle of length 1 s).

One method which can overcome the temporal and spatial resolution limitations of 4D-flow is computational fluid dynamics (CFD). CFD has been shown to provide highly detailed hemodynamic information in numerous cardiovascular diseases, with the ability to accurately characterise complex hemodynamic patterns beyond velocity alone (Bordones et al., 2018; Fanni et al., 2023; Sabry et al., 2023; Tsubata et al., 2019; 2023). Within PAH, CFD studies have indicated that TAWSS is lower in PAH patients compared to healthy volunteers (Tang et al., 2012; Pillalamarri et al., 2021). Abnormal TAWSS magnitudes and patterns are known to influence endothelial cell function in the lining of vessel walls (Chiu et al., 2004; 2005; 2007; Hastings et al., 2007; Wang et al., 2013; Arshad et al., 2021; Armour et al., 2024). With the knowledge that PAH leads to arterial stiffening and remodelling, there is a desire and opportunity to better understand how PAH driven hemodynamics contribute to disease progression. Additionally, understanding the connection between TAWSS and PAH may allow for improved diagnosis and treatment development.

While CFD provides more confidence in exact magnitudes of hemodynamics parameters, prior studies that have analysed pulmonary hemodynamics, including those that have specifically identified TAWSS patterns, in PAH patients and the interpretation of their results are limited by the choice of model set up parameters, especially boundary conditions applied with idealised flat or parabolic profiles (Tang et al., 2012; Bordones et al., 2018; Pillalamarri et al., 2021; Fanni et al., 2023). Sabry et al. (2023) significantly improved on these prior studies by implementing 2D inlet velocity profiles, in six subjects, derived from patient-specific 2D-flow MRI scans. Within the region of interest, the main pulmonary artery, being adjacent to the ejection of blood from the right ventricle through the pulmonary valve, flow patterns in the main pulmonary artery are highly non-uniform and individual in each patient. The application of a simplified flat or parabolic inlet velocity profile (as has often been applied in previous CFD models of PAH) will likely be to miss distinct flow features in the pulmonary arteries. While 2D profiles are a step closer towards physiological accuracy, studies in the ascending aorta (anatomically analogous to the main pulmonary artery) have shown that only a patient-specific 3D inlet velocity profile (derived from 4D-flow MRI data) can accurately model hemodynamics (Pirola et al., 2017; Armour et al., 2020). Additionally, Armour et al. (2020) demonstrated the influence of stroke volume on TAWSS, with a 25% reduction in stroke volume leading to a 35% reduction in TAWSS–an important finding to keep in mind when interpreting CFD results based on a population average pulmonary flow profile.

The use of patient-specific 4D-flow MRI based CFD models to derived high resolution hemodynamic information has been demonstrated and validated in various diseased and healthy arterial models (Armour et al., 2022; Yin et al., 2024; Wang et al., 2024). Thus, the aim of this study is to apply a 4D-flow MRI based CFD methodology to a PAH cohort, to accurately evaluate TAWSS and assess any correlation to RHC measured mean PAP. To the best of our knowledge, this is the first study to apply a fully patient-specific hemodynamic workflow by implementing a 3D inlet velocity profile.

## 2 Materials and methods

Seven patients (P1-7) diagnosed and treated for PAH at Hammersmith Hospital, London were included in this study. Collection and use of all data had approval from the UK National Health Service Health Research Authority. All patients were undergoing regular checkups and monitoring (including, RHC, echocardiogram, CT) as standard of care, with clinical information being collected as part of the TRIPHIC database (IRAS ID: 210461). Additionally, the patients were recruited to undergo a 4D-flow MRI scan alongside their standard scans (IRAS ID: 280472). The resolution of the 4D-flow scans for all patients were in the range of 2 mm × 2.375–2.625 mm × 2.375–2.625 mm. Among the seven patients, the time between the RHC and 4D-flow MRI scan varied between one and 30 days (average of 9 days).

**FIGURE 1:**
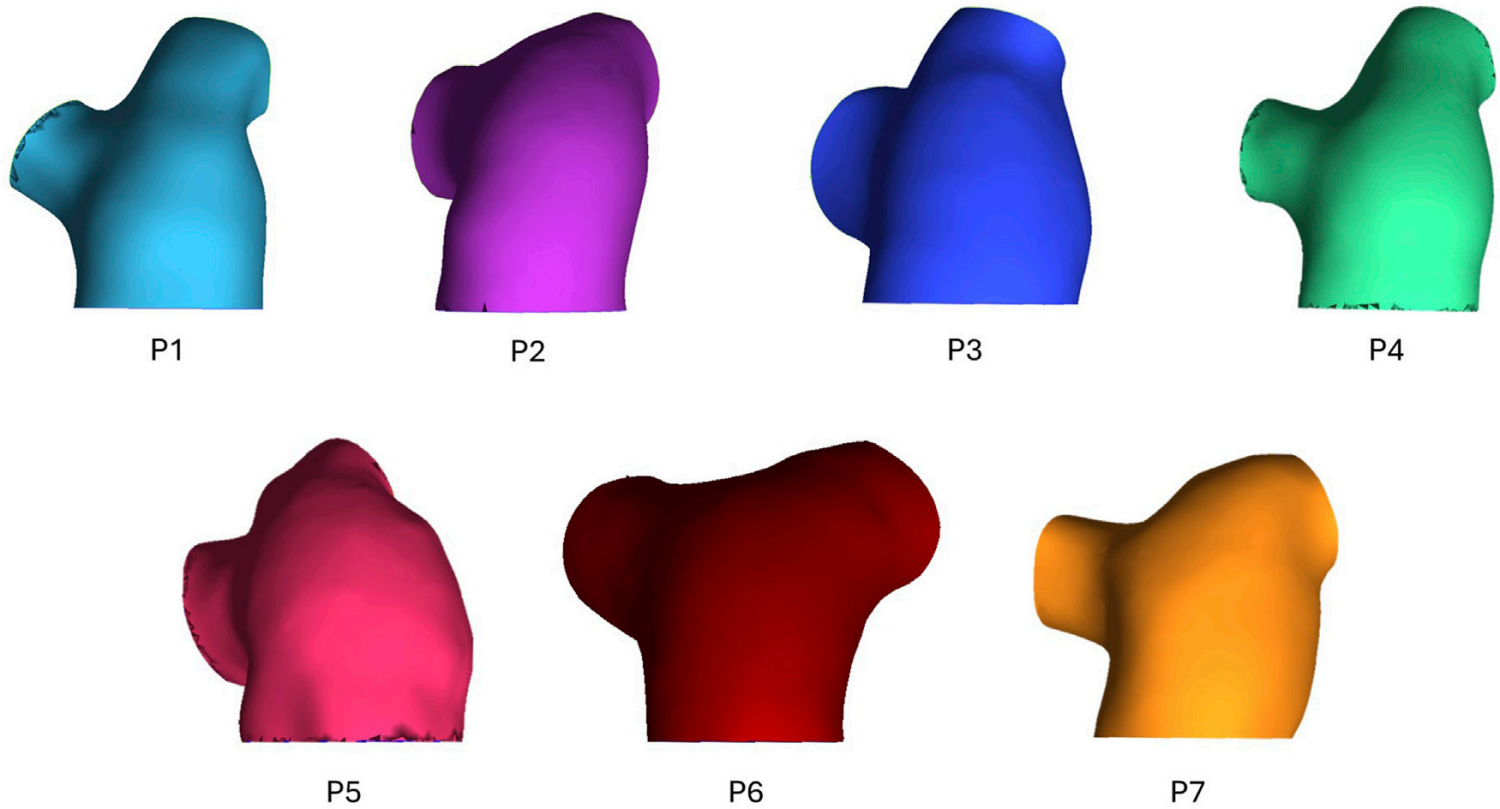
Geometric models of seven PAH patients (P1-7) reconstructed from 4D-flow MRI data.

3D geometric models of the pulmonary artery, main bifurcation, and right (RPA) and left pulmonary artery (LPA) branches were reconstructed from the 4D-flow MRI data. Using Mimics (Materialize v22), 2D masks of the pulmonary artery were segmented on each slice of all three velocity phase images as well as the magnitude images. Individually, this would allow for four separate 3D models to be generated, with different sections of the pulmonary artery segmented depending on the dominant direction of flow in each region. Therefore, by combing all layers of the four masks through the inbuilt addition ‘Boolean Operations’, a final complete 3D geometry of the whole pulmonary artery was reconstructed–the geometric models for all seven patients can be seen in Figure 1. Geometries were meshed in ICEM (Ansys, v22), containing a hexahedral core and 10 prism layers at the wall. Mesh sensitivity tests were performed on one exemplar geometry to ensure mesh independent solutions–mesh parameters were deemed acceptable when velocity and wall shear converged below 5%. The chosen mesh parameters were then set for all subsequent geometries to ensure consistency. Final meshes varied between 0.46–1.09 million elements.

Using in-house python codes (Saitta, 2022; Saitta et al., 2023), 3D inlet velocity profiles were extracted from each patient’s 4D-flow MRI scan and applied as the inlet boundary condition. 3-element Windkessel (3EWK) models were tuned for the right and left pulmonary artery in each model following the methodology reported by Pirola et al. (2017). To make the 3EWK parameters patient-specific, a total branch resistance must be estimated from average pressure and flow values for each patient. Average flow to the left and right pulmonary artery was measured from 4D-flow MRI data, while average pressure for each patient was extracted from echocardiogram. Peak pressure was first estimated using the simplified Bernoulli’s equation: peak PAP [mmHg] = 4·v^2^ + RAP, where v is the peak tricuspid valve velocity measured during the echocardiogram, and RAP is an estimate of the right atrial pressure, set by the clinician at the time of echocardiography depending on inferior vena cava diameter and respiratory variation. Mean PAP was then estimated as mean PAP = 0.61 peak PAP + 2 (Chemla et al., 2024). As the model was developed as a diagnostic tool it was assumed that the RHC invasive pressure readings were not available for tuning the 3EWK parameters. Therefore, the parameters were implemented in the models as first calculated with the input patient-specific average 4D-flow-derived flow and echocardiogram-derived pressure values. All simulations were run for a minimum of three cardiac cycles to ensure the outlet flow and pressure values being calculated by the 3EWK model became periodic, with a variation of less than 1% between the final two cycles. Table 1 reports key flow information of the inlet velocity profile as well as the 3EWK parameters for all patients.

**TABLE 1:** Key flow information *extracted from 4D-flow MRI and **measured from right heart catheter, and 3EWK parameters for patients P1-7.

Parameter	P1	P2	P3	P4	P5	P6	P7
Cardiac Cycle [s]*	0.95	0.75	0.92	0.65	0.83	0.64	0.69
Stroke Volume [mL]*	95	41	184	45	84	76	36
% inlet flow to RPA*	62	44	51	51	61	52	69
Mean diagnostic PAP [mmHg]**	44	55	27	68	52	45	49
RPA 3-element Windkessel parameters							
R1 [x10^7^ Pa s m^−3^]	1.15	0.94	0.44	1.23	0.70	1.34	1.59
R2 [x10^8^ Pa s m^−3^]	0.71	2.26	0.17	2.42	1.84	0.88	1.70
C [x10^−9^ m^3^ Pa^−1^]	9.12	3.18	35.04	2.95	3.93	7.36	4.03
LPA 3-element Windkessel parameters							
R1 [x10^7^ Pa s m^−3^]	1.16	0.90	0.81	1.25	1.09	1.21	1.88
R2 [x10^8^ Pa s m^−3^]	0.88	2.27	0.31	2.52	2.71	1.35	2.14
C [x10^−9^ m^3^ Pa^−1^]	7.53	3.18	19.36	2.83	2.66	5.10	3.22

RPA, right pulmonary artery; LPA, left pulmonary artery; 3EWK, 3-element Windkessel; R1 – proximal resistance; R2 – distal resistance; C – compliance.

Previous literature has reported that transition to turbulence in the aorta occurs at a critical value of 150α, where α is the Womersley number defined as 2π/period (Kousera et al., 2012). As similar data has not been reported for the pulmonary artery, the same criterion was applied in this study due to the anatomical and functional similarities of the aorta and pulmonary artery. Thus, peak Reynolds number was calculated for all models and was found to be below the critical value for transition to turbulence. Therefore, flow was assumed to be and modelled as laminar in all cases.

The non-Newtonian behaviour of the blood was captured through the Quemada model, with model parameters taken from literature (Mimouni, 2016). All simulations were run with a timestep of 0.005 s in CFX (Ansys, v22) for a minimum of three cardiac cycles to ensure a periodic solution. The final cycle was used for analysis, with post-processing done in EnSight (Ansys, v22). Qualitative and quantitative comparisons of velocity and flow fields between CFD and 4D-MRI data were first conducted for validation. Further analysis of the numerical results was then conducted to evaluate TAWSS values and patterns. The CFD calculated TAWSS values were then correlated against RHC measured mean PAP, as this is the key parameter required for diagnosis of PAH. Any correlation identified between TAWSS and mean PAP would thus indicate a potential non-invasive computational biomarker for PAH.

## 3 Results

### 3.1 Validation of CFD against 4D-flow MRI

**FIGURE 2:**
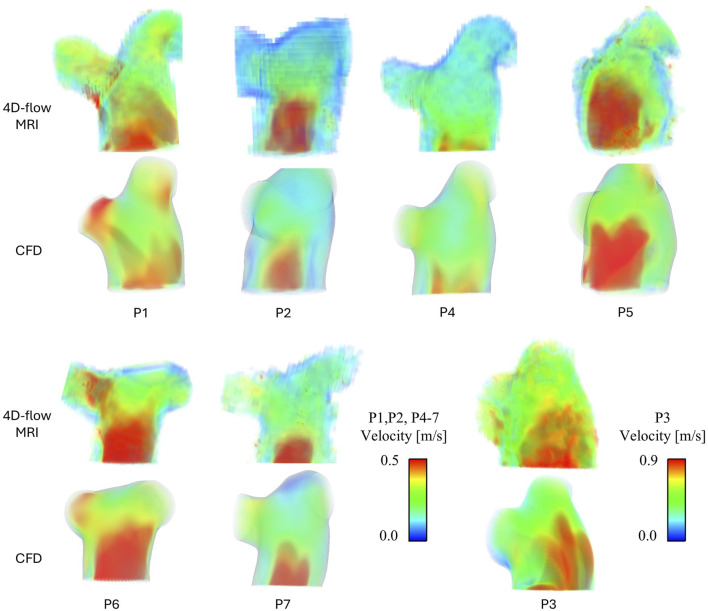
Peak systolic velocity fields for all patients, extracted from 4D-flow MRI data and modelled with CFD. P3 has been separated due to a different velocity scale.

Figure 2 presents peak systolic velocity fields from 4D-flow MRI data and CFD models. Across all patients the CFD predicted velocities are well matched to the *in-vivo* 4D-flow data. All patients presented with a clear velocity jet entering the main pulmonary artery, however, the variation in jet distribution and size is clear, further highlighting the importance of a patient-specific inlet velocity profile. P3 observed higher velocities through the pulmonary artery compared to all patients, represented by the increased velocity scale (upper limit of 0.9 m/s vs. 0.5 m/s). This is expected due to P3’s high stroke volume (183 mL). Not only do the CFD models capture well the intricate non-uniform velocity jets in the main pulmonary artery that vary significantly across patients, but also, the unique distribution of flow to the LPA and RPA in each patient is clearly observed. For example, local regions of increased velocities match well between CFD and 4D-flow data in the LPA and RPA of P1, and the RPA of P6 and P7.

To quantitatively validate the CFD models, the percentage of flow reporting to each branch and the mean peak systolic velocity on a plane in the centra of the main pulmonary artery were measured from both the 4D-flow MRI scans and CFD simulations–Table 2 reports this data. On average CFD velocities in the main pulmonary artery were within 10% of 4D-flow MRI velocities (range 5%–19%), and all CFD models simulated the LPA/RPA distribution within 3.5% of the 4D-flow measurement. The strong quantitative and qualitative agreement in velocity fields between the CFD and 4D-flow MRI data gives confidence in the following step of evaluating derivative hemodynamic parameters from the CFD data.

**TABLE 2:** Comparison of hemodynamic parameters measured from 4D-flow MRI and calculated from CFD simulation.

Parameter	P1	P2	P3	P4	P5	P6	P7
% inlet flow to RPA – 4D-flow	0.62	0.44	0.51	0.51	0.61	0.52	0.69
% inlet flow to RPA – CFD	0.62	0.45	0.52	0.51	0.63	0.51	0.70
% inlet flow to LPA – 4D-flow	0.38	0.56	0.49	0.49	0.39	0.48	0.31
% inlet flow to LPA – CFD	0.38	0.55	0.48	0.49	0.37	0.49	0.30
Mean peak systolic MPA velocity [m/s] – 4D-flow	0.327	0.207	0.690	0.174	0.266	0.317	0.255
Mean peak systolic MPA velocity [m/s] –CFD	0.349	0.185	0.582	0.207	0.278	0.353	0.267

RPA, right pulmonary artery; LPA, left pulmonary artery; MPA, main pulmonary artery.

### 3.2 CFD predicted WSS

**FIGURE 3:**
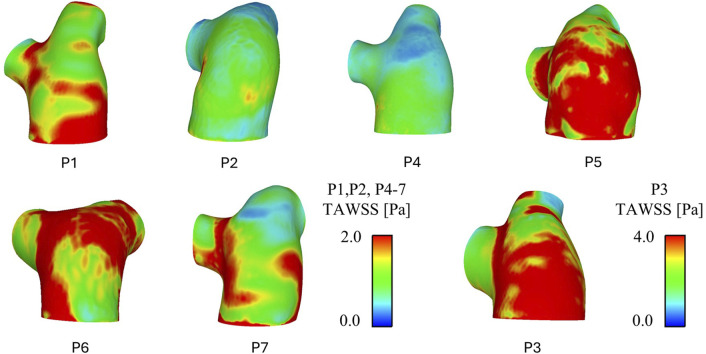
CFD predicted time-average wall shear stress (TAWSS) patterns for all patients. P3 has been separated due to a different TAWSS scale.

Figure 3 shows TAWSS contours for all seven patients. As with the velocity results, varying TAWSS patterns are observed across the cohort. P2 and P4 observed the lowest TAWSS throughout the pulmonary artery; P1 and P5-7 exhibited similar levels of TAWSS, with unique localisations indicating the locations where the pulmonary valve velocity jets were impinging on the wall and diverting to the LPA and RPA. Again, in line with the velocities, P3 observed significantly higher TAWSS than the other patients–this would be expected given the increased velocities in volume of similar size.

Spatially averaged TAWSS values over the whole geometry (main pulmonary artery and initial segment of the LPA and RPA) and in the main pulmonary artery alone (proximal to the bifurcation) are reported in Table 3. TAWSS ranged from 0.63 to 3.62 across the seven patients, with P2 seeing the lowest TAWSS values and P3 the highest. All patients saw higher TAWSS when taking the LPA and RPA into account, however the magnitude of influence of the inclusion of the LPA and RPA varied: P2, P4, and P5 saw less than 0.2 Pa increase, P1, P6, and P7 an increase of 0.3–0.4 Pa, and P3 and increase of 1 Pa.

**TABLE 3:** Spatially averaged, over two regions of the geometric model, TAWSS for patients P1-7.

TAWSS [Pa] Location
	P1	P2	P3	P4	P5	P6	P7
Main pulmonary artery + LPA + RPA	1.62	0.76	3.62	0.71	1.77	2.01	1.34
Main pulmonary artery	1.25	0.63	2.56	0.67	1.60	1.61	1.03

RPA, right pulmonary artery; LPA, left pulmonary artery; TAWSS, time-averaged wall shear stress.

**FIGURE 4:**
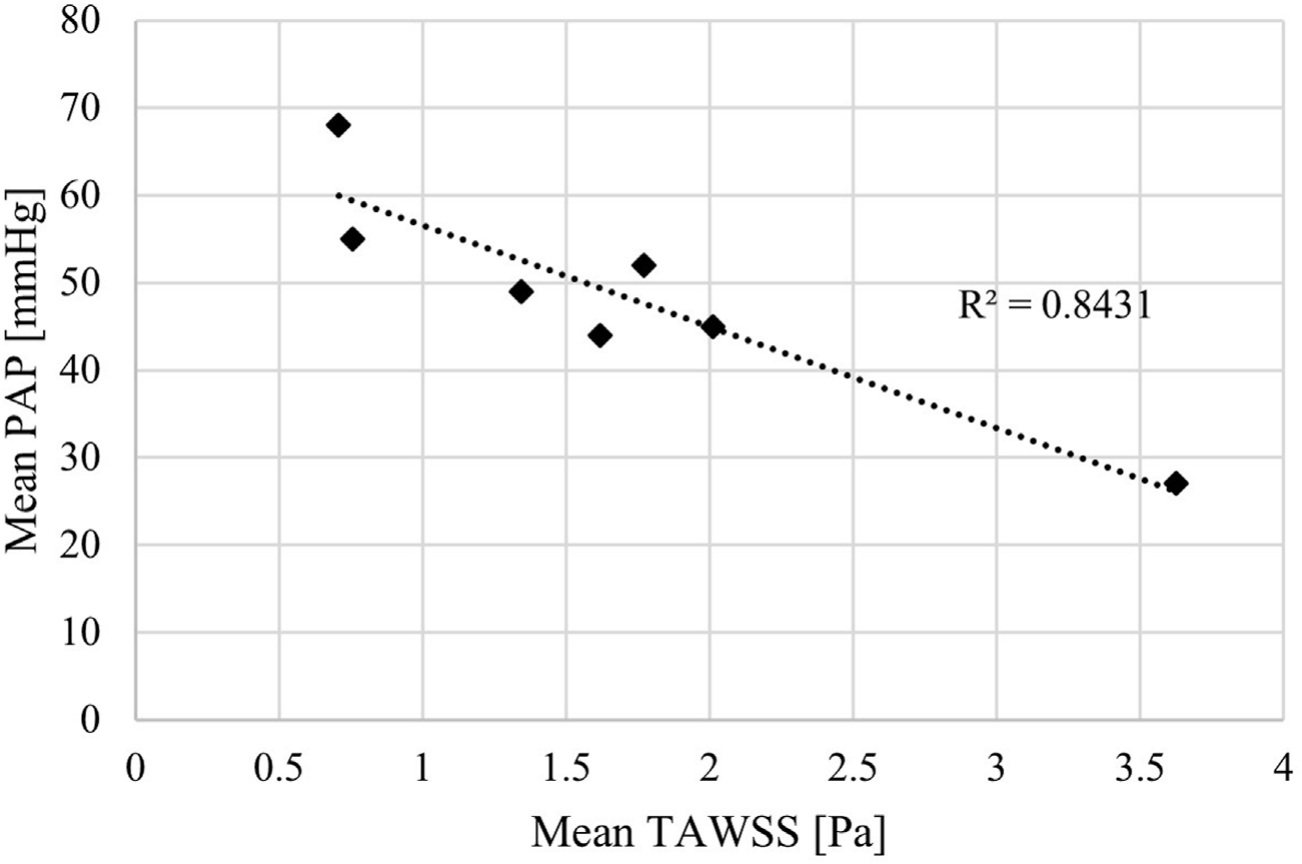
Mean time-averaged wall shear stress (TAWSS) estimated by patient-specific CFD models correlated with mean pulmonary arterial pressure (PAP) measured by right heart catheter.

Both sets of TAWSS values (main pulmonary artery alone, and then with the addition of LPA and RPA) were tested for correlations with invasively measured RHC PAP (values reported in Table 1). A linear correlation was observed for mean PAP vs. TAWSS in the main pulmonary artery + LPA + RPA region, with R^2^ = 0.84 (Figure 4). Considering the main pulmonary artery segment alone, the R^2^ was 0.77.

## 4 Discussion

PAH is a challenging disease to diagnose, treat, and manage. Symptoms can often be subtle and, as PAH requires a technical and invasive RHC procedure for diagnosis, referral to a specialist centre is required. With this, patients can be interacting with doctors for years before referral (Kiely et al., 2019a). This extended period between symptom onset and diagnosis leads to worse patient outcomes and higher healthcare costs (Lawrie et al., 2024). Yet a wealth of data is available on these patients due to the numerous interactions with their local healthcare system. This provides an opportunity to develop novel non-invasive diagnostic methods based on this data to better identify patients at risk of PAH and improve early diagnosis numbers.

In this study, a methodology to build patient-specific 4D-flow MRI based CFD models, previously developed and validated in aortic dissection (Armour et al., 2022), was applied to a pilot cohort of PAH patients. Pulmonary artery hemodynamics of seven patients were modelled and analysed to identify hemodynamics parameters that are linked to mean PAP (the key diagnostic parameter for PAH).

The velocity fields for all patients shown in Figure 2 were evaluated in detail. The variation in pulmonary valve velocity jet and resulting TAWSS contours demonstrates how individual pulmonary hemodynamics are, and the importance of developing patient-specific CFD models. Within the cohort the size, distribution, and magnitude of the main jet varies, as well as the distribution to the LPA and RPA. From Figure 2, P1 and P6 observe significantly different velocities between their own LPA and RPA, yet P1sees higher TAWSS in the RPA where velocities are higher while P6 sees higher TAWSS in the LPA where velocities are lower. These unbalanced velocities and TAWSS between the LPA and RPA in P1 and P6 correspond to the relatively uneven LPA/RPA flow split measured from the 4D-flow data as reported in Table 1. However, P7 saw the most uneven LPA/RPA flow split, with 69% of inlet flow reporting to the RPA, yet there were less drastic variations in velocities.

The strong match between the CFD simulation velocity fields and 4D-flow MRI data allowed for CFD calculated TAWSS patterns, and importantly magnitudes, to be further interpreted and linked to patient-specific clinical data. This resulted in the identification of a linear correlation between TAWSS and mean PAP of 0.84. Given the sample size of seven patients, this cannot be classed as statistically significant, however, it is a clear indication of a potential computational biomarker of PAH. This aligns with the findings of Schafer et al. who evaluated TAWSS directly from 4D-flow MRI. Importantly, the presented methodology of using CFD to evaluate TAWSS opens up the potential to focus on the difficult area of patients with PAPs near the 20 mmHg diagnostic threshold. With more confidence in exact magnitudes of TAWSS estimated from CFD (as opposed to 4D-flow MRI) larger cohort studies can identify incremental thresholds that better distinguish PAP values and PAH severity.

Interestingly, the correlation between TAWSS and mean PAP slightly reduced to 0.77 when TAWSS was evaluated in the main pulmonary artery alone (excluding the beginning of the LPA and RPA). This may suggest that the bifurcation, both in terms of geometry, alignment, and possibly biomechanical properties is related to high pressure. Exactly how and where in the development of the disease this is related requires further study. In studies that have focused on vortical flow (Cerne et al., 2022; Sabry et al., 2023) vortices were seen to gather and dissipate around the bifurcation area. Such vortical flow would influence TAWSS, further strengthening the hypothesis that the pulmonary artery bifurcation is influential in hemodynamics relating to PAH.

As WSS is dependent on velocity gradient, both vessel diameter and stroke volume can significantly impact WSS values due to their influence on velocity. Therefore, correlations of WSS against both vessel diameter and stroke volume (as a surrogate of velocity) were evaluated. Vessel diameter showed a weak R^2^ value of 0.36 against mean PAP, suggesting that vessel diameter alone cannot predict mean PAP. Stroke volume reported a R^2^ value of 0.69 against mean PAP. This is a stronger correlation than diameter yet lower still than the R^2^ value of 0.84 for the CFD derived TAWSS. This suggests that while stroke volume is potentially more closely linked to PAP, there are more factors influencing PH than stroke volume alone–TAWSS also encapsulates variations in the 3D geometry of the pulmonary artery.

Finally, as mentioned in the introduction, TAWSS has been estimated directly from 4D-flow MRI in previous studies. The limitation with these 4D-flow values arises due to the low spatial resolution. For example, a 2D plane cut in the main pulmonary artery of patient P1 contained approximately 4700 and 260 mesh elements for the CFD model and 4D-flow MRI, respectively. The significantly enhanced resolution of the CFD model allows for flow patterns and velocity gradients to be captured in greater detail, particularly near the wall. In the 4D-flow MRI data small deviations in velocities will be lost due to the large voxels, resulting in lower velocity gradients and therefore lower TAWSS values.

As a comparison, TAWSS was estimated directly from the 4D-flow MRI data on planes placed in the MPA, RPA, and LPA using cvi42 (Circle Cardiovascular Imaging Inc.). The average and range of these values among the seven patients are reported in Table 4, along with the corresponding CFD values. The 4D-flow values are substantially lower than the CFD values and cover a smaller range across all three planes (4D-flow MRI: 0.04–0.19 Pa vs. CFD: 0.45–3.59 Pa). Among the seven patients, the 4D-flow MRI values showed no correlation with RHC measured mean PAP (R^2^ < 0.1). However, this is a small sample set and evaluation in a larger patient cohort will allow for more confident conclusions. Future work will also look to quantitatively compare TAWSS distributions across the entire pulmonary artery wall.

**TABLE 4:** Averaged (and range of) TAWSS among the seven patients (P1-7) on planes placed in the main (MPA), right (RPA), and left pulmonary artery (LPA) for the CFD model and 4D-flow MRI data.

TAWSS [Pa] Location
	CFD average (range)	4D-flow MRI average (range)
MPA	1.72 (0.78–3.59)	0.11 (0.07–0.15)
RPA	1.52 (0.60–2.66)	0.11 (0.06–0.19)
LPA	1.43 (0.45–3.01)	0.08 (0.04–0.13)

A key limitation of this work is the small number of patients included. In this case, this pilot study cohort has been used to identify potential computational biomarkers for PAH diagnosis and severity classification, and further work is ongoing to validate these findings in a significantly larger cohort. In this future work, healthy and non-PAH cardiovascular disease controls will be assessed to overcome the limitation of the current study being limited to PAH only. In terms of the computational model, the assumption of a rigid wall will impact simulated hemodynamics. However, it is known that increased pulmonary artery stiffness comes with PAH (Schafer et al., 2016), thus the impact on WSS values of the rigid wall will likely be reduced, compared to a healthy model. Furthermore, modelling wall motion significantly increase computational complexity, and reduces clinical deployment feasibility. Provided methodology is consistent, suitable rigid model WSS thresholds identified in a larger cohort study can still be used to stratify patients. Finally, the extent of the geometric model was limited to the main pulmonary artery and initial section of the LPA and RPA due to the scan region of the 4D-flow MRI. In future work, segmentation from anatomical CT or MRI will be incorporated to extend the geometric model to the second branch of the pulmonary tree to fully assess TAWSS in the LPA and RPA.

The results of this study demonstrate a clear indication that WSS is linked to pulmonary artery pressure in PAH patients. Specifically, higher pulmonary artery pressure is associated with lower TAWSS. These findings lay the groundwork for future work to develop non-invasive computational models for improved early diagnosis and risk stratification in PAH.

## Data Availability

All processed data, that complies with data governance and ethics, supporting the conclusions of this article will be shared by authors upon reasonable request.
